# Higher Adherence to Plant-Based Diet Lowers Type 2 Diabetes Risk among High and Non-High Cardiovascular Risk Populations: A Cross-Sectional Study in Shanxi, China

**DOI:** 10.3390/nu15030786

**Published:** 2023-02-03

**Authors:** Ying Zhang, Yaqing Meng, Junbo Wang

**Affiliations:** 1 Department of Nutrition and Food Hygiene, School of Public Health, Peking University, Beijing 100191, China; 2 Shanxi Provincial Center for Disease Control and Prevention, Taiyuan 030012, China; 3 Beijing Key Laboratory of Toxicological Research and Risk Assessment for Food Safety, Peking University, Beijing 100191, China

**Keywords:** plant-based diets, high cardiovascular risk, type 2 diabetes, cross-sectional study, Shanxi Province

## Abstract

This study aimed to investigate the association between the plant-based diet index (PDI) score and T2D risk among residents of Shanxi Province, China, and explore whether the association was influenced by different levels of cardiovascular risk. A total of 50,694 participants aged 35–75 years were recruited between 2017 and 2019, and they were further divided into the high cardiovascular risk population (HCRP; *n* = 17,255) and the non-high cardiovascular risk population (non-HCRP; *n* = 33,439). The PDI was calculated based on food frequency from a food frequency questionnaire (FFQ). Incident T2D was defined based on elevated plasma glucose (≥7 mmol/L) or hypoglycemic medicine use. We investigated the association of the PDI andT2D risk using a two-level generalized estimating equation and restricted cubic splines model. The results showed that quartile 4 of the PDI indicated significantly reduced T2D risk in the total population (OR: 0.83; 95% CI: 0.75–0.92), HCRP (OR: 0.80; 95% CI: 0.71–0.91), and non-HCRP (OR: 0.80; 95% CI: 0.74–0.87) compared with corresponding quartile 1 (OR = 1). In stratified analysis, the negative associations between PDI and T2D risk were stronger in the total population with the elderly (age > 60 years), BMI < 24, and men, and in the non-HCRP with men and BMI 24–28, and in the HCRP with the elderly and BMI < 24 than those with corresponding subgroups (*p*_interaction_ < 0.05). Linear curves were observed for the total population and non-HCRP, but an L-shaped association was observed for the HCRP. Therefore, our results suggest that higher PDI scores may effectively attenuate the T2D risk in the Chinese population and non-HCRP, and a beneficial association of PDI with T2D risk was observed in the HCRP at a certain threshold level. Longitudinal studies and intervention trials are required to validate our study findings.

## 1. Introduction

With rapid economic growth, accompanied by poor lifestyle habits and unhealthy dietary patterns, the global prevalence of diabetes, mainly type 2 diabetes, shows an increasing trend, with 537 million in 2021 expected to increase to 783 million in 2045 [1]. In addition, T2D is correlated with an increased risk of cardiovascular disease. The incidence of T2D in people at high cardiovascular risk (51.6%), characterized by higher blood pressure and lipids, is more than four times that in the general population (11.2%) in China [2,3]. High cardiovascular risk populations with disorders in glucose, blood pressure or lipid regulation are more likely to experience premature death than non-high cardiovascular risk people. Owing to its serious complications and tremendous socioeconomic burden [4,5], T2D remains a major public health problem that needs to be addressed. People at high cardiovascular risk are the main intervention targets to prevent and delay T2D because of its prevalence and harmfulness.

Diet is one of the modifiable risk factors with regard to the occurrence of T2D [6]. Furthermore, a plant-based diet can significantly prevent or mitigate T2D and cardiovascular disease, and replace drugs and surgery in some cases [7]; thus, it is a recommendation in the 2015 Dietary Guidelines for Americans. However, the plant-based diet varies considerably in terms of food categories and intake across groups, cultures, and regions [8]. The current dietary pattern methods include a priori analysis, posteriori analysis, reduced rank regression (RR) and LASSO regression [9]. The priori analysis, known as the index method, is easy to calculate and objective and has received much attention.

A plant-based diet index (PDI) is one kind of common indices created by Satija et al. in 2016 that scores animal foods negatively and measures plant foods positively, which highlights the role of plant foods versus other indices. In addition, this index is more acceptable because it reflects gradually decreasing rather than completely excluding animal food relative to a vegetarian diet. Furthermore, existing studies suggest that higher PDI scores indicate a reduced risk of T2D [5,10,11,12,13,14,15,16,17,18]. However, there are some gaps in previous studies on the relationship between a plant-based diet and T2D risk. First, studies on the correlation between PDI and T2D have presented inconclusive results, which are negative [5,10,11,12,13,14,15,16,17,18] or absent [19,20,21]. Second, the majority of research participants were from Western populations, whose dietary patterns and metabolic responses are different from those of Asian populations [22]. Asian people have a higher consumption of grains, which influence blood glucose metabolism [23]. To our knowledge, there are only three studies on related Chinese populations [14,18,24]: the Singapore Chinese Health study (SCHS), the Henan Rural Cohort Study, and the China Nutrition and Health Survey (CNHS). However, these studies have their own limitations. For example, the SCHS and CNHS were initiated in 1993 or 2004, when dietary patterns and the disease spectrum were significantly different compared with the present. In addition, the association between baseline information on dietary intake and T2D was conducted by the traditional COX model in the SCHS and CNHS, which may have led to dietary pattern measurement errors and attenuation of the true association. The results of the Henan study were difficult to directly extrapolate to other populations, as the participants were farmers with special diets and lifestyles. Finally, the association between PDI and T2D risk among people at high cardiovascular risk has not been reported. Hence, there is a need to conduct more research.

Thus, we attempted to evaluate the association between a plant-based diet and the risk of T2D in a Chinese population and wondered whether the relationship was influenced by cardiovascular risk.

## 2. Materials and Methods

### 2.1. Study Design and Participants

As part of the China Patient-Centered Evaluative Assessment of Cardiac Events Million Persons Project (MPP), this study (MPP-Shanxi) was conducted in Shanxi Province, China, between March 2017 and May 2019. The study has been described by Lu et al. [25]. Briefly, a total of 60,006 eligible community-dwelling participants aged 35–75 years from four cities and six counties were screened using a cluster sampling strategy with physical measurements, a blood test, and a questionnaire. All participants provided informed consent, and the study protocol was approved by the Ethics Committee of Fu Wai Hospital, Chinese Academy of Medical Sciences (Code: 2014-574). We report this study based on the guidelines of Strengthening the Reporting of Observational Studies in Epidemiology (STROBE).

**Figure 1:**
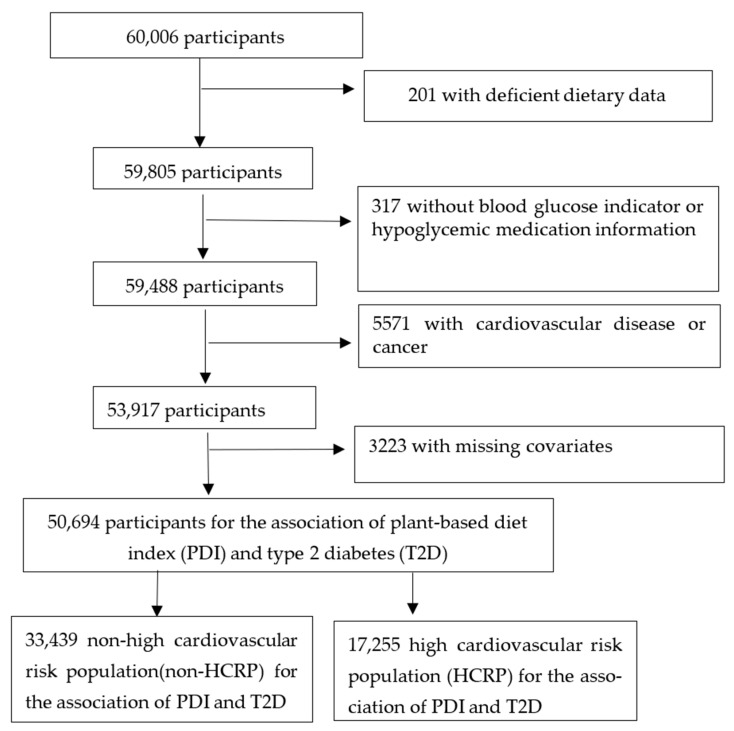
Flow chart of participant selection.

From 60,006, we excluded participants with at least one blank item on the FFQ (*n* = 201); lack of blood glucose indicator or hypoglycemic medication (*n* = 317), missing covariates, including marital status, smoking status, physical activity, drinking status, waist circumference, occupation, household income, body mass index (BMI) (*n* = 3233), cancer (except nonmelanoma skin cancer) and cardiovascular disease (*n* = 5571). Finally, 50,694 participants were included (Figure 1).

### 2.2. Determining the High Cardiovascular Risk Population (HCRP)

The high cardiovascular risk population (HCRP) was defined by any of the following criteria: high blood pressure (SBP ≥ 160 mmHg or DBP ≥ 100 mmHg), high blood lipids (LDL ≥ 4.14 mmol/L or HDL < 0.78 mmol/L), and a 10-year CVD risk ≥ 20% [26]. Participants were divided into the non-high cardiovascular risk population (non-HCRP; *n* = 33,439) and high cardiovascular risk population (HCRP; *n* = 17,255) (Figure 1).

### 2.3. Dietary Assessment and Plant-Based Diet Index Score

The plant-based dietary pattern evaluation was conducted using the 12-item FFQ, as shown in Table S1. This FFQ, with good reliability and validity, was the same as the one used in the China Kadoorie Biobank study [27]. The items were divided into plant foods (whole grains, fruits, fresh vegetables, refined grains, preserved vegetables) and animal foods (eggs, seafood, meat, poultry, milk and dairy), with slight differences from previous studied dietary categories [16]. Alcoholic beverages were not included in the plant-based diet but were adjusted in the analysis as a covariate. The options for food frequency were “rarely or never”, “1–3 days per month”, “≥1 day per week”, and “every day”. Intake of plant foods such as fruits, vegetables and grains less than 3 days per week was defined as deficiency, whereas intake of meat every day was determined as excess.

Based on the previous literature [16,28,29], the PDI was calculated by food frequency, with scores of 5, 4, 3 and 1, respectively, for consumption of plant foods every day, ≥1 time per week, 1–3 times per month, or rarely or never (positive scores); for consumption of animal foods, the scores were 1, 3, 4 and 5, respectively (reverse scores) [28]. PDI values ranging from 12 to 60 for each participant indicated the sum of the scores of all food categories/groups. The PDI was divided into four groups according to quartiles from smallest to largest (Q1, Q2, Q3 and Q4). In our analysis, PDI was categorized into quartiles (categorical variables) and 1 standard deviation (SD) (continuous variable).

### 2.4. Determination of Type 2 Diabetes

Based on the diagnostic criteria of the American Diabetes Association (ADA) [30], the incidence of T2D was based on a fasting glucose concentration ≥7.0 mmol/L or the use of a hypoglycemic drug.

Blood samples were taken from the fingertip after ≥8 h of fasting. A rapid electrochemical glucose analyzer (Bene Check BK6–20 M Multi-Monitoring System, Suzhou Puchuntang Biotechnology Co., Suzhou, China) was used to test the glucose concentration at the local health service centers.

### 2.5. Covariates

Covariates related to sociodemographic information, lifestyle and behaviors, physical indicators, and medical history were obtained through structured questionnaires by trained study staff. Sociodemographic information included age (standardization, continuous variable), sex, marital status (married, cohabiting, other), education (high school or above, other), occupation (farmer, retired, other), geographic region (urban, rural), and annual household income (<RMB 50,000, ≥RMB 50,000). Lifestyle and behaviors included smoking status (never, other), drinking status (≥2 days/week, other), and physical activity (≥1 day/week, other) over the past year. Physical indicators included body mass index (BMI) (calculated by the formula: BMI = weight (kg)/height (m)^2^, normal <24, overweight 24–28, obese >28 kg/m^2^) and waist circumference (central obesity (TC ≥ 90 cm for men and TC ≥ 85 cm for women) and normal). Height to the nearest 0.1 cm and weight to the nearest 0.1 kg were measured without shoes and heavy clothes. Waist circumference was measured using a leather ruler at the midpoint between the anterior superior iliac crest and the lower border of the 12th rib. There were hypertension (considered as SBP ≥ 140 mmHg or DBP ≥ 90 mmHg measured, self-reported use of drugs) and dyslipidemia (defined as TC ≥ 6.22 mmol/L or TG ≥ 2.32 mmol/L or HDL-C < 1.04 mmol/L or LDL-C ≥ 4.12 mmol/L tested, self-reported use of drugs) in the medical history (classified as “yes” or “no”) [31,32]. The details have been described by Zhu et al. [32].

### 2.6. Statistical Analysis

Characteristics of participants grouped by PDI quartiles were expressed as mean ± standard deviation (SD) for continuous variables or number (percentage) for categorical variables. Analysis of variance (ANOVA) and the chi-square test (χ^2^) were used to test the differences in continuous and categorical variables, respectively.

To estimate the association of the PDI quartiles with the risk of T2D, we adjusted for multiple covariates in the three models using a two-level generalized estimating equation model [14] taking community as one level. Model 1 was age and sex. Model 2 included marital status, education, household income, geographic region, occupation, physical activity, tobacco smoking, alcohol drinking, waist circumference, dyslipidemia, and hypertension. Model 3 added BMI on the basis of Model 2. Adjusting for Model 3, we tested the risk of T2D for each SD after PDI standardization. To test for underlying nonlinear correlations, we examined the trend of the median of each PDI quartile in adjusting for Model 3 as a continuous variable. To verify the robustness of the results, we performed two sensitivity analyses. In one analysis, we interpolated the missing covariates using the multiple imputation of chained equations method. We performed this analysis again using the optimal imputed data with the highest reliability analysis score. In the other analysis, to illustrate the imbalanced probability of self-selection into PDI quartile groups, we calculated the multigroup propensity scores between PDI and covariates through a generalized boosted model (GBM). The optimal number of GBM iterations was determined by the es.mean method. Differences between PDI categories were balanced because the absolute standardized mean difference (ASMD) was less than 0.2 with 10,000 iterations, as shown in Figures S1–S6. T2D risk was obtained by logistic regression of the weighted data by the propensity score of inverse probability of treatment weighting (IPTW).

To examine the dose–response relationship, the shape of the PDI association was analyzed in a restricted cubic splines model with automatic knots selected by minimum AIC standard adjusting for Model 3. To assess the effect of a plant-based diet on T2D risk in certain subgroups, we performed stratified analyses based on participants’ sex, age (≤60 and >60 years), and BMI (<24, 24–28, and >28 kg/m^2^). To investigate whether the relationship was influenced by cardiovascular risk, we performed all of the above analyses for the HCRP and non-HCRP groups. All data were analyzed using SPSS software version 22.0 and the twang and survey packages in R version 3.6.3, and a two-tailed *p* value of <0.05 was considered obviously significant.

## 3. Results

### 3.1. Participant Characteristics

Our study included 50,694 participants (29,871 women and 20,823 men) with a mean age of 55.3 ± 9.67 years after exclusions. The total incidence of T2D among them was 15.1% (7654/50,694). The average PDI of the participants was 46.0 ± 3.85. Table 1 shows the characteristics of participants grouped by quartiles of PDI. The rates of T2D in groups Q1, Q2, Q3, and Q4 were 16.6, 14.9, 14.0, and 14.1%, respectively. Participants with higher PDI scores tended to be older and women, had lower household incomes, had lower education levels, lived in rural regions, and had little physical activity, but had lower rates of drinking, smoking, and T2D, and consumed higher amounts of vegetables, fruits, and whole grains, and lower amounts of meat compared with those in PDI Q1 (lowest quartile) (*p* < 0.001). The HCRP consumed more vegetables relative to the non-HCRP in the individual quartile group (Table S2).

### 3.2. Association between Plant-Based Diet Index and Type 2 Diabetes

Table 2 reports the odds ratios (ORs) and 95% confidence intervals (CIs) for T2D in the total population, non-HCRP, and HCRP based on quartiles and 1 SD of PDI. There was a significant downwards trend in T2D risk with increasing PDI quartiles among the total population, non-HCRP, and HCRP, adjusted for all models (*p*_trend_ < 0.05). A higher PDI was negatively associated with the risk of T2D among the total population (OR: 0.82, 95% CI, 0.76–0.89), non-HCRP (OR: 0.80, 95% CI, 0.72–0.88), and HCRP (OR: 0.81, 95% CI, 0.72–0.90), adjusted for all models. Meanwhile, each 1 SD increase in the PDI significantly resulted in 9, 9 and 10% reductions in the risk of T2D in the total population (OR: 0.91, 95% CI, 0.89–0.93), non-HCRP (OR: 0.91, 95% CI, 0.88–0.94), and HCRP (OR: 0.90, 95% CI, 0.87–0.94), respectively.

Two sensitivity analyses demonstrated similar results among these populations. In the analysis with missing covariates interpolated (Table S3), the risk of T2D was separately reduced by 19% (before imputation, 18%), 12% (before imputation, 20%), and 19% (before imputation, 19%) in Q4 among the total population (OR: 0.81,95% CI, 0.72–0.91), non-HCRP (OR: 0.78,95% CI, 0.69–0.88), and HCRP (OR: 0.81, 95% CI, 0.68–0.96) compared with the individual Q1 group. In the propensity score weighted study (Table S4), the PDI Q4 also indicated decreased T2D risk versus Q1 in the total population (OR: 0.82,95% CI, 0.76–0.90), HCRP (OR: 0.82, 95% CI, 0.73–0.92) and non-HCRP (OR: 0.82, 95% CI, 0.74–0.92).

### 3.3. Dose–Response Relationship between Plant-Based Diet Index and Type 2 Diabetes

**Figure 2:**
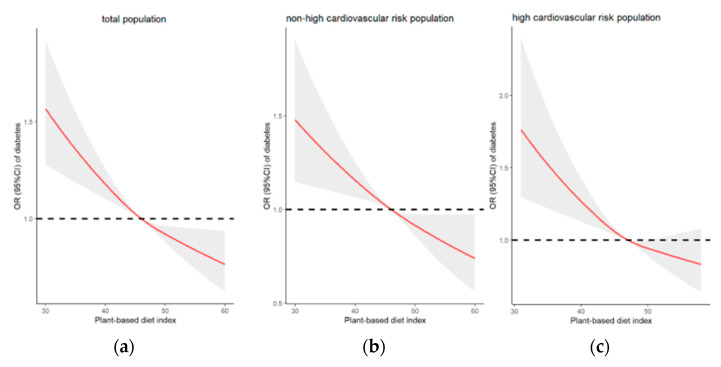
Multivariable-adjusted odds ratios (ORs) and 95% confidence intervals (CIs) for T2D risk based on the continuous PDI in three different populations adjusted for a series of covariates in Model 3. (**a**) Association between PDI and T2D risk in total population (**b**) Association between PDI and T2D risk in non-high cardiovascular risk population (non-HCRP) (**c**) Association between PDI and T2D risk in high cardiovascular risk population (HCRP). The shaded area represents 95% confidence intervals. The red lines indicate the multivariable-adjusted ORs for T2D risk. The dotted lines are the median value as a reference group, corresponding to an OR of 1.0.

Figure 2 depicts the dose—response relationship between the continuous PDI and T2D incidence adjusted for a series of variables in Model 3 among the total population, non-HCRP, and HCRP using restricted cubic splines with automatic knots selected by minimum AIC. There was a linear trend of decreasing T2D risk with increasing PDI among the total population and non-HCRP (Figure 2a,b). An L-shaped curve was observed for the correlation between risk of T2D and PDI in HCRP, showing that PDI was inversely associated with T2D risk when PDI was less than 50, while a PDI over 50 was not significantly associated with T2D risk compared with the median value (Figure 2c). All *p* values for nonlinearity were greater than 0.05.

### 3.4. Subgroup Analyses

**Figure 3:**
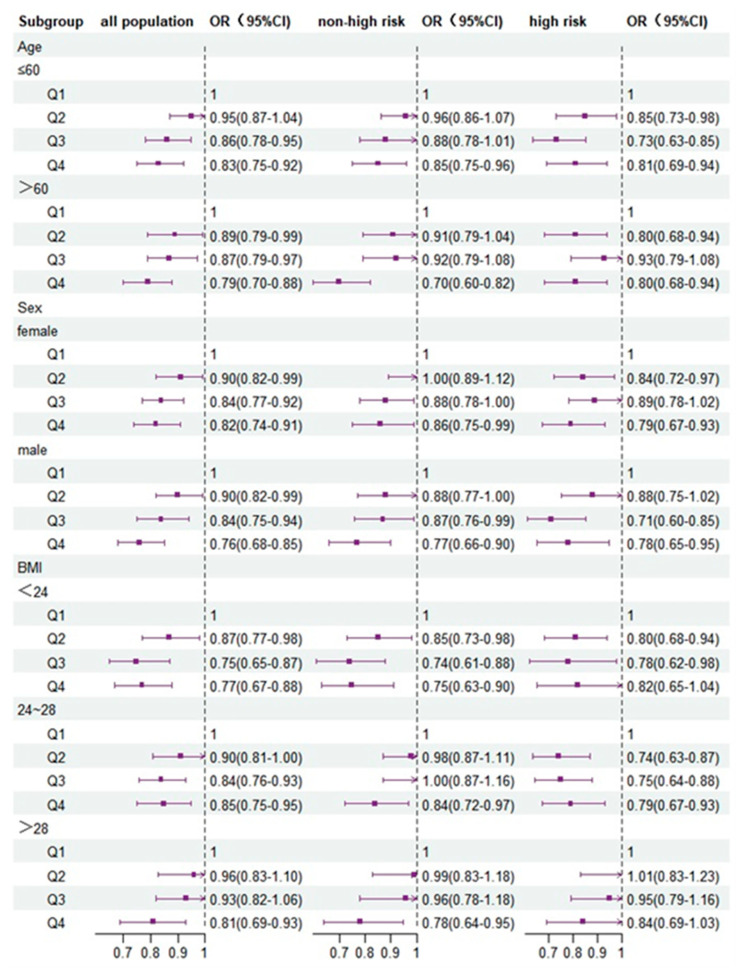
Odds ratios (ORs) and 95% confidence intervals (CIs) for T2D risk according to PDI quartiles stratified by age, sex and BMI adjusted for a series of covariates in Model 3 compared with PDI Q1. Non-high risk, non-high cardiovascular risk population. High risk, high cardiovascular risk population.

Figure 3 shows that the associations among subgroups stratified by sex, age, and BMI in the total population, non-HCRP, and HCRP varied after adjusting for variables in Model 3. The negative associations between PDI and T2D risk were stronger in the total population with the elderly (age > 60 years), BMI < 24, and men, and in non-HCRP with men and BMI 24–28, and in HCRP with the elderly and BMI < 24 than those with corresponding subgroups (p_interaction_ < 0.05).

## 4. Discussion

In this study, the PDI Q4 was associated with a lower T2D risk compared with Q1 in the total population, non-HCRP and HCRP. The results were confirmed by two sensitivity analyses. Restricted cubic splines between PDI values and ORs of T2D risk showed a negative linear tendency for the total population and non-HCRP and an L-shaped association for HCRP. Subgroup analyses discovered that the decreased risk of T2D was more significant in the total population with the elderly (age > 60 years), BMI < 24 and men, and in non-HCRP with men, BMI 24–28, and in HCRP with the elderly and BMI < 24 than those with corresponding subgroups.

Prior studies on the relationship between a plant-based diet and diabetes risk have revealed contradictory results. In agreement with our results, some studies indicated that a higher PDI score was beneficial for lowering the risk of T2D [5,13,16,17]. A recent meta-analysis recorded an inverse effect of higher PDI on diabetes risk across subgroups, determined by various population characteristics and robustness in sensitivity analysis (RR, 0.77; 95% CI, 0.71–0.84) [33]. Satija et al. reported that a higher PDI value was associated with a 49% lower risk of T2D in US health professionals in three large prospective cohort studies [4]. This negative relationship between PDI and T2D risk was validated in Dutch [13] and Korean [34] populations. In addition, higher PDI values were associated with lower T2D risk in Chinese individuals in the SCHS, Henan Rural Cohort Study, and CHNS [14,18,24]. However, unlike our results, the Korean Genome and Epidemiology Study (KoGES) and Puerto Rico Cohort study demonstrated that PDI was not associated with T2D incidence [17,19]. These differences may be due to dietary habits, adjusted covariates, study inclusion criteria, and nondietary lifestyle risk factors.

A linear decreasing trend in the risk of T2D with increasing PDI was shown in the total population and non-HCRP, which was consistent with all previous studies [14,16,24,33]. An L-shaped curve was determined for HCRP. Some studies have found that vegetables have no effect on incident T2D [35]. More vegetable intake in HCRP relative to non-HCRP (Table S2) weakened the association between higher PDI scores and T2D risk, which may account for the L-shaped association for T2D. In addition, there may also be a reverse causal relationship in which HCRP with T2D and chronic disease (hypertension and dyslipidemia) are preferred to plant-based patterns. To the best of our knowledge, this study is the first to investigate the association of PDI with T2D risk among the high cardiovascular risk population. Therefore, it is difficult to find literature for comparison. However, the diet in this study, as measured by the PDI, is somewhat similar to other generally accepted plant-based dietary patterns, such as the Mediterranean diet (Med-Diet) and Dietary Approaches to Stop Hypertension (DASH), which have been proven to lessen T2D risk in people at high cardiovascular risk [36,37]. For example, with the Med-Diet, supplementation with extra virgin olive oil significantly decreased fasting glucose compared with the control diet group for participants at high cardiovascular risk [38]. For those in the highest quintile on the Med-Diet, the risk of new diabetes was reduced by 35% (OR: 0.65; 95% CI: 0.49–0.85) compared with the lowest quintile among 8,291 patients with new myocardial infarction at baseline in the large GISSI-Prevenzione study [39]. Greater adherence to DASH was significantly associated with lower HbA1c and glucose concentrations among Spanish adults aged 65 years at high cardiovascular risk in the Prevencion con Diet a Mediterranea (PREDIMED)-Plus trial [37].

The stratified analysis showed that the decreased association between PDI and T2D risk was stronger among individuals with a lower BMI than a higher BMI, because a plant-based diet can influence the risk of T2D by modulating BMI [40,41], but BMI is also an independent risk factor for T2D. The results also indicated a sex difference in PDI values owing to differences in dietary patterns. The plant-based dietary index is more sensitive to T2D risk among men who prefer to consume more animal foods or saturated fats [42]. A plant-based diet has a positive effect on the risk of T2D in older people, because they are more receptive to adopting such a diet.

Some mechanisms can account for the beneficial effect of a plant-based diet on the risk of T2D when considering plant and animal foods. Whole grains, fruits, and vegetables are rich in dietary fiber and antioxidants. Dietary fiber can regulate glucose metabolism by decreasing blood glucose absorption and postponing gastric emptying [18,43]. Antioxidants can promote insulin secretion and sensitivity, leading to lower postprandial blood glucose [44]. High amounts of polyphenols and antioxidants have been proven to reduce the risk of T2D in people at high cardiovascular risk [45]. On the other hand, a lower intake of animal food reduces branched-chain and aromatic amino acids, which is detrimental to glucose metabolism [46,47], and decreases saturated fats, leading to obesity and T2D [48]. Hence, a plant-based diet can decrease the risk of T2D by regulating blood glucose metabolism and controlling weight.

This study is the first to explore the relationship between a plant-based diet and T2D risk in a population with different levels of cardiovascular risk by a generalized estimating equation model with two levels. The advantages of this study include the large community sample, adjusted comprehensive covariates, different demographic characteristics, and the community aggregation of risk factors. However, there are also several limitations. First, this was a cross-sectional study in which we could not distinguish the causal relationship between a plant-based diet and the risk of T2D. Second, the PDI was not calculated by food consumption but by a nonquantitative FFQ that had been proven to be reliable and valid to assess plant foods [49], and frequency of intake is more important than portion size of fruits and vegetables for Asians. Third, the frequency of dietary intake was self-reported by participants, which may have led to recall bias. Fourth, it was not possible to exclude the effect of residual confounding because of the nature of an observational study. Fifth, the vast majority of our data were based on a qualitative questionnaire. Finally, our results are probably most applicable to northern populations and should not be generalized to other populations.

## 5. Conclusions

Our results showed that higher PDI scores were associated with a reduced risk of T2D in the Chinese population and a certain threshold level of PDI can reduce the risk of T2D in people at high cardiovascular risk. Our findings support the use of a plant-based diet to prevent T2D among Chinese individuals. It is necessary to implement longitudinal studies and intervention trials to investigate the long-term influence of a plant-based diet on the risk of T2D among individuals with different levels of cardiovascular risk.

## Figures and Tables

**Table 1:** Characteristics of participants grouped by quartiles of plant-based diet index.

	Q1 (*n* = 16,965)	Q2 (*n* = 10,480)	Q3 (*n* = 14,479)	Q4 (*n* = 8970)	*p*
PDI	27–44	45–46	47–49	50–60	
Glucose (mmol/L) ^a^	6.00 ± 1.60	5.91 ± 1.45	5.87 ± 1.45	5.88 ± 1.42	<0.001
Age (years) ^a^	54.7 ± 9.89	54.9 ± 9.67	55.5 ± 9.55	56.3 ± 9.23	<0.001
Sex, *n* (%)					<0.001
Women	9305 (54.8)	5922 (56.5)	8664 (60.7)	5980 (66.7)	
Men	7660 (45.2)	4558 (43.5)	5615 (39.3)	2990 (33.3)	
Geographic region, *n* (%)					<0.001
Urban	9264 (54.6)	3976 (37.9)	3599 (25.2)	1766 (19.7)	
Rural	7701 (45.4)	6504 (62.1)	10,680 (74.8)	7204 (80.3)	
Education level, *n* (%)					<0.001
High school or above	5862 (34.6)	2803 (26.7)	2980 (20.9)	1404 (15.7)	
Other	11,103 (65.4)	7677 (73.3)	11,299 (79.1)	7566 (84.3)	
Marital status, *n* (%)					<0.001
Married/cohabiting	16,142 (95.1)	9944 (94.9)	13,458 (94.3)	8416 (93.8)	
Other	823 (4.9)	536 (5.1)	821 (5.7)	554 (6.2)	
Occupation, *n* (%)					<0.001
Farmer	7334 (43.2)	5811 (55.4)	9307 (65.2)	6514 (72.6)	
Retired	4102 (24.2)	2005 (19.1)	2240 (15.7)	1287 (14.3)	
Other	5529 (32.6)	2664 (25.4)	2732 (19.1)	1169 (13)	
Household income, *n* (%)					<0.001
<RMB 50,000	14,467 (85.3)	9423 (89.9)	13,142 (92)	8466 (94.4)	
≥RMB 50,000	2498 (14.7)	1057 (10.1)	1137 (8)	504 (5.6)	
Physical activity, *n* (%)					
≥1 day/week	5806 (34.2)	3171 (30.3)	3632 (25.4)	2320 (25.9)	
Other	11,159 (65.8)	7309 (69.7)	10,647 (74.6)	6650 (74.1)	
BMI, *n* (%)					<0.001
24	6358 (37.5)	3679 (35.1)	4825 (33.8)	2847 (31.7)	
24–28	7496 (44.2)	4641 (44.3)	6351 (44.5)	4044 (45.1)	
≥28	3111 (18.3)	2160 (20.6)	3103 (21.7)	2079 (23.2)	
Alcohol drinking, *n* (%)					<0.001
≥2 days/week	1517 (8.9)	713 (6.8)	929 (6.5)	515 (5.7)	
Other	15,448 (91.1)	9767 (93.2)	13,350 (93.5)	8455 (94.3)	
Tobacco smoking, *n* (%)					<0.001
Never	12,762 (75.2)	7845 (74.9)	11,098 (77.7)	7201 (80.3)	
Other	4203 (24.8)	2635 (25.1)	3181 (22.3)	1769 (19.7)	
Waist circumference, *n* (%)					<0.001
Obese	6070 (35.8)	3576 (34.1)	4648 (32.6)	2839 (31.6)	
Normal	10,895 (64.2)	6904 (65.9)	9631 (67.4)	6131 (68.4)	
T2D, *n* (%)					<0.001
Yes	2822 (16.6)	1560 (14.9)	2004(14)	1268 (14.1)	
No	14,143 (83.4)	8920 (85.1)	12,275 (86)	7702 (85.9)	
Fruit, *n* (%)					<0.001
1–3 days/month	478 (27.8)	1791 (17.1)	2196 (15.4)	1163 (13)	
≥1day/week	12,257 (72.2)	8689 (82.9)	12,083 (84.6)	7807 (87)	
Vegetables, *n* (%)					<0.001
1–3 days/month	1818 (10.7)	526 (5)	380 (2.7)	101 (1.1)	
≥1 day/week	15,147 (89.3)	9954 (95)	13,899 (97.3)	8869 (98.9)	
Grain, *n* (%)					<0.001
1–3 days/month	4646 (27.4)	1763 (16.8)	1,571 (11)	524 (5.8)	
≥1 day/week	12,319 (72.6)	8717 (83.2)	12,708 (89)	8446 (94.2)	
Meat, *n* (%)					<0.001
Every day	2549 (15)	514 (4.9)	499 (3.5)	264 (2.9)	
6 days/week	14,416 (85)	9966 (95.1)	13,780 (96.5)	8706 (97.1)	

Note: T2D, type 2 diabetes. Q, quartile. BMI, body mass index. Q1, Q2, Q3 and Q4 are quartiles of PDI from low to high. Categorical variables are expressed as numbers (%), and analyzed by the chi-square test among quartile groups. ^a^ Continuous values are exhibited as the mean ± standard deviation (SD), and distinguished by analysis of variance (ANOVA) among quartile groups. *p* < 0.001 indicates a significant difference among quartile groups.

**Table 2:** Odds ratios (ORs) and 95% confidence intervals (CIs) for incident type 2 diabetes according to the plant-based diet index score.

Group	Score Range	*n*/*N*	Model 1	Model 2	Model 3
			OR (95% CI)	OR (95% CI)	OR (95% CI)
Total population					
Q1	27–44	2822/16,965	1.00	1.00	1.00
Q2	45–46	1560/10,480	0.87 (0.81–0.93) *	0.90 (0.84–0.96) *	0.90 (0.83–0.96) *
Q3	47–49	2004/14,279	0.79 (0.74–0.84) *	0.83 (0.78–0.89) *	0.83 (0.78–0.89) *
Q4	50–60	1268/8970	0.78 (0.72–0.84) *	0.83 (0.77–0.90) *	0.82 (0.76–0.89) *
*p*_trend_			<0.001	<0.001	<0.001
1SD			0.90 (0.87–0.91) *	0.91 (0.89–0.94) *	0.91 (0.89–0.93) *
Non-HCRP					
Q1	27–43	1404/9573	1.00	1.00	1.00
Q2	44–46	1340/10,129	0.88 (0.82–0.96)	0.94 (0.87–1.03)	0.94 (0.87–1.02)
Q3	47–48	761/6125	0.82 (0.74–0.90) *	0.90 (0.82–0.99) *	0.90 (0.82–0.99) *
Q4	49–60	838/7612	0.70 (0.63–0.76) *	0.80 (0.73–0.89) *	0.80 (0.72–0.88) *
*p*_trend_			<0.001	<0.001	<0.001
1SD			0.87 (0.84–0.90) *	0.92 (0.88–0.95) *	0.91 (0.88–0.94) *
HCRP					
Q1	27–45	1294/5839	1.00	1.00	1.00
Q2	46–47	649/3616	0.77 (0.69–0.85) *	0.83 (0.74–0.93) *	0.83 (0.74–0.92) *
Q3	48–49	696/4042	0.72 (0.65–0.80) *	0.82 (0.74–0.91) *	0.82 (0.73–0.91) *
Q4	50–60	672/3758	0.73 (0.66–0.81) *	0.81 (0.73–0.91) *	0.81 (0.72–0.90) *
*p*_trend_			0.002	0.010	<0.001
1SD			0.86 (0.83–0.90) *	0.91 (0.87–0.94) *	0.90 (0.87–0.94) *

Note: Q, quartile. *n*, the number of people with T2D and *N*, the total number of people. Non-HCRP, non-high cardiovascular risk population. HCRP, high cardiovascular risk population. * Values indicate significant differences between the labelled group and Q1. Model 1: adjusted for age (year, continuous) and sex. Model 2: adjusted for Model 1 + tobacco smoking (never, other), alcohol drinking (≥2 days/week, other), physical activity (≥1 day/week, other), marital status (married, cohabiting, other), waist circumference (central obesity, normal), geographic region (rural, urban), occupation (farmer, retired, other), education (high school or above, other), household income (<RMB 50,000, ≥RMB 50,000), hypertension (yes, no), and dyslipidemia (yes, no). Model 3: adjusted for Model 2 + BMI (<24, 24–28, ≥28 kg/m^2^). *p*_trend_, probability was calculated by putting the median of each quartile of PDI in adjustment for covariates in Model 3 as a continuous variable. 1 SD in the total population, non-HCRP and HCRP were 3.85, 3.89, and 3.67, respectively.

## Data Availability

The data on which our study is based are not publicly available.

## Supplementary Materials

The following supporting information can be downloaded at: https://www.mdpi.com/article/10.3390/nu15030786/s1, Table S1: Classification of food items in the Early Screening and Comprehensive Intervention Program for high-risk Population of Cardiovascular Disease Study Table S2: Characteristics of vegetables according to quartile of PDI among high cardiovascular risk and non-high cardiovascular risk populations. Table S3: Odds ratios (ORs) and 95% confidence intervals (CIs) for type 2 diabetes odds ratios (ORs) and 95% confidence intervals (CIs) for type 2 diabetes according to the plant-based diet index after interpolating missing covariates. Table S4: Odds ratios (ORs) and 95% confidence intervals (CIs) for type 2 diabetes according to the plant-based diet index after weighting the data by multinomial propensity score. Figure S1: The balance measures of interest at different interactions in the total population. Figure S2: The balance measures of interest at different interactions in the high cardiovascular risk population. Figure S3: The balance measures of interest at different interactions in the non-high cardiovascular risk population. Figure S4: The absolute standardized mean differences (AMSDs) of covariates in the total population. Figure S5: The absolute standardized mean differences (AMSDs) of covariates in the high cardiovascular risk population. Figure S6: The absolute standardized mean differences (AMSDs) of covariates in the non-high cardiovascular risk population.

## References

[1] Ogurtsova K., Guariguata L., Barengo N.C., Ruiz P.L.D., Sacre J.W., Karuranga S., Sun H., Boyko E.J., Magliano D.J. (2022). IDF diabetes Atlas: Global estimates of undiagnosed diabetes in adults for 2021. Diabetes Res. Clin. Pract..

[2] Lu J., Lu Y., Yang H., Bilige W., Li Y., Schulz W., Masoudi F.A., Krumholz H.M. (2019). Characteristics of high cardiovascular risk in 1.7 million Chinese adults. Ann. Intern. Med..

[3] Li Y., Teng D., Shi X., Qin G., Qin Y., Quan H., Shi B., Sun H., Ba J., Chen B. (2020). Prevalence of diabetes recorded in mainland China using 2018 diagnostic criteria from the American Diabetes Association: National cross sectional study. BMJ.

[4] Chatterjee S., Khunti K., Davies M.J. (2017). Type 2 diabetes. Lancet.

[5] Chen Z., Drouin-Chartier J.-P., Li Y., Baden M.Y., Manson J.E., Willett W.C., Voortman T., Hu F.B., Bhupathiraju S.N. (2021). Changes in plant-based diet indices and subsequent risk of type 2 diabetes in women and men: Three US prospective cohorts. Diabetes Care.

[6] Aune D., Norat T., Romundstad P., Vatten L.J. (2013). Whole grain and refined grain consumption and the risk of type 2 diabetes: A systematic review and dose–response meta-analysis of cohort studies. Eur. J. Epidemiol..

[7] Matsumoto S., Beeson W.L., Shavlik D.J., Siapco G., Jaceldo-Siegl K., Fraser G., Knutsen S.F. (2019). Association between vegetarian diets and cardiovascular risk factors in non-Hispanic white participants of the Adventist Health Study-2. J. Nutr. Sci..

[8] Storz M.A. (2022). What makes a plant-based diet? a review of current concepts and proposal for a standardized plant-based dietary intervention checklist. Eur. J. Clin. Nutr..

[9] Liu Q., Shi Z.M., Yu C.-Q. (2021). Progress on statistical methods in evaluating Dietary Patterns. Acta Nutr. Sin..

[10] Laouali N., Shah S., MacDonald C.-J., Mahamat-Saleh Y., El Fatouhi D., Mancini F., Fagherazzi G., Boutron-Ruault M.-C. (2021). BMI in the Associations of Plant-Based Diets with Type 2 Diabetes and Hypertension Risks in Women: The E3N Prospective Cohort Study. J. Nutr..

[11] Daneshzad E., Jahangir F., Heshmati J., Larijani B., Surkan P.J., Azadbakht L. (2021). Associations between plant-based dietary indices and dietary acid load with cardiovascular risk factors among diabetic patients. Int. J. Diabetes Dev. Ctries..

[12] Chen Z., Qian F., Liu G., Li M., Voortman T., Tobias D.K., Ley S.H., Bhupathiraju S.N., Li L.-J., Chavarro J.E. (2021). Prepregnancy plant-based diets and the risk of gestational diabetes mellitus: A prospective cohort study of 14,926 women. Am. J. Clin. Nutr..

[13] Chen Z., Zuurmond M.G., van der Schaft N., Nano J., Wijnhoven H.A.H., Ikram M.A., Franco O.H., Voortman T. (2018). Plant versus animal based diets and insulin resistance, prediabetes and type 2 diabetes: The Rotterdam Study. Eur. J. Epidemiol..

[14] Yang X., Li Y., Wang C., Mao Z., Chen Y., Ren P., Fan M., Cui S., Niu K., Gu R. (2021). Association of plant-based diet and type 2 diabetes mellitus in Chinese rural adults: The Henan Rural Cohort Study. J. Diabetes Investig..

[15] Wang H., Huang L., Lin L., Chen X., Zhong C., Li Q., Li N., Gao D., Zhou X., Chen R. (2021). The overall plant-based diet index during pregnancy and risk of gestational diabetes mellitus: A prospective cohort study in China. Br. J. Nutr..

[16] Satija A., Bhupathiraju S.N., Spiegelman D., Chiuve S.E., Manson J.E., Willett W., Rexrode K.M., Rimm E.B., Hu F.B. (2017). Healthful and unhealthful plant-based diets and the risk of coronary heart disease in US adults. J. Am. Coll. Cardiol..

[17] Kim J., Giovannucci E. (2022). Healthful Plant-Based Diet and Incidence of Type 2 Diabetes in Asian Population. Nutrients.

[18] Chen G.C., Koh W.P., Neelakantan N., Yuan J.M., Qin L.Q., van Dam R.M. (2018). Diet Quality Indices and Risk of Type 2 Diabetes Mellitus: The Singapore Chinese Health Study. Am. J. Epidemiol..

[19] Flores A.C., Heron C., Kim J.I., Martin B., Al-Shaar L., Tucker K.L., Gao X. (2021). Prospective Study of Plant-Based Dietary Patterns and Diabetes in Puerto Rican Adults. J. Nutr..

[20] Amini M.R., Shahinfar H., Djafari F., Sheikhhossein F., Naghshi S., Djafarian K., Clark C.C., Shab-Bidar S. (2021). The association between plant-based diet indices and metabolic syndrome in Iranian older adults. Nutr. Health.

[21] Kim H., Lee K., Rebholz C.M., Kim J. (2021). Association between unhealthy plant-based diets and the metabolic syndrome in adult men and women: A population-based study in South Korea. J. Nutr..

[22] Micha R., Khatibzadeh S., Shi P., Andrews K.G., Engell R.E., Mozaffarian D. (2015). Global, regional and national consumption of major food groups in 1990 and 2010: A systematic analysis including 266 country-specific nutrition surveys worldwide. BMJ Open.

[23] Hopping B.N., Erber E., Grandinetti A., Verheus M., Kolonel L.N., Maskarinec G. (2010). Dietary fiber, magnesium, and glycemic load alter risk of type 2 diabetes in a multiethnic cohort in Hawaii. J. Nutr..

[24] Chen B., Zeng J., Qin M., Xu W., Zhang Z., Li X., Xu S. (2022). The Association Between Plant-Based Diet Indices and Obesity and Metabolic Diseases in Chinese Adults: Longitudinal Analyses From the China Health and Nutrition Survey. Front. Nutr..

[25] Lu J., Xuan S., Downing N.S., Wu C., Li L., Krumholz H.M., Jiang L. (2016). Protocol for the China PEACE (Patient-centered Evaluative Assessment of Cardiac Events) million persons project pilot. BMJ Open.

[26] Cournot M., Taraszkiewicz D., Cambou J.-P., Galinier M., Boccalon H., Hanaire-Broutin H., Chamontin B., Carrié D., Ferrières J. (2009). Additional prognostic value of physical examination, exercise testing, and arterial ultrasonography for coronary risk assessment in primary prevention. Am. Heart J..

[27] Yu C., Shi Z., Lv J., Guo Y., Bian Z., Du H., Chen Y., Tao R., Huang Y., Chen J. (2017). Dietary patterns and insomnia symptoms in Chinese adults: The China Kadoorie Biobank. Nutrients.

[28] Chen H., Shen J., Xuan J., Zhu A., Ji J.S., Liu X., Cao Y., Zong G., Zeng Y., Wang X. (2022). Plant-based dietary patterns in relation to mortality among older adults in China. Nat. Aging.

[29] Zhu A., Chen H., Shen J., Wang X., Li Z., Zhao A., Shi X., Yan L., Zeng Y., Yuan C. (2022). Interaction between plant-based dietary pattern and air pollution on cognitive function: A prospective cohort analysis of Chinese older adults. Lancet Reg. Health-West. Pac..

[30] Association A.D. (2010). Diagnosis and classification of diabetes mellitus. Diabetes Care.

[31] Zhu J., Gao R., Zhao Y., Lu G., Zhao D., Li J. (2016). Guidelines for the prevention and treatment of dyslipidemia in adults in China (2016 Revision). J. Chin. Inst. Eng..

[32] Zhu F., Qin Y., Bi Y., Su J., Cui L., Luo P., Du W., Miao W., Wang J., Zhou J. (2021). Fresh vegetable and fruit consumption and carotid atherosclerosis in high-cardiovascular-risk population: A cross-sectional study in Jiangsu, China. Cad. Saúde Pública.

[33] Qian F., Liu G., Hu F.B., Bhupathiraju S.N., Sun Q. (2019). Association between plant-based dietary patterns and risk of type 2 diabetes: A systematic review and meta-analysis. JAMA Intern. Med..

[34] Kim H., Lee K., Rebholz C.M., Kim J. (2020). Plant-based diets and incident metabolic syndrome: Results from a South Korean prospective cohort study. PLoS Med..

[35] Neuenschwander M., Ballon A., Weber K.S., Norat T., Aune D., Schwingshackl L., Schlesinger S. (2019). Role of diet in type 2 diabetes incidence: Umbrella review of meta-analyses of prospective observational studies. BMJ.

[36] Salas-Salvadó J., Bulló M., Estruch R., Ros E., Covas M.-I., Ibarrola-Jurado N., Corella D., Arós F., Gómez-Gracia E., Ruiz-Gutiérrez V. (2014). Prevention of diabetes with Mediterranean diets: A subgroup analysis of a randomized trial. Ann. Intern. Med..

[37] Glenn A.J., Hernández-Alonso P., Kendall C.W., Martínez-González M.Á., Corella D., Fitó M., Martínez J.A., Alonso-Gómez Á.M., Wärnberg J., Vioque J. (2021). Longitudinal changes in adherence to the portfolio and DASH dietary patterns and cardiometabolic risk factors in the PREDIMED-Plus study. Clin. Nutr..

[38] Estruch R., Martínez-González M.A., Corella D., Salas-Salvadó J., Ruiz-Gutiérrez V., Covas M.I., Fiol M., Gómez-Gracia E., López-Sabater M.C., Vinyoles E. (2006). Effects of a Mediterranean-style diet on cardiovascular risk factors: A randomized trial. Ann. Intern. Med..

[39] Mozaffarian D., Marfisi R., Levantesi G., Silletta M.G., Tavazzi L., Tognoni G., Valagussa F., Marchioli R. (2007). Incidence of new-onset diabetes and impaired fasting glucose in patients with recent myocardial infarction and the effect of clinical and lifestyle risk factors. Lancet.

[40] Satija A., Malik V., Rimm E.B., Sacks F., Willett W., Hu F.B. (2019). Changes in intake of plant-based diets and weight change: Results from 3 prospective cohort studies. Am. J. Clin. Nutr..

[41] Toumpanakis A., Turnbull T., Alba-Barba I. (2018). Effectiveness of plant-based diets in promoting well-being in the management of type 2 diabetes: A systematic review. BMJ Open Diabetes Res. Care.

[42] Vitale M., Masulli M., Cocozza S., Anichini R., Babini A.C., Boemi M., Bonora E., Buzzetti R., Carpinteri R., Caselli C. (2016). Sex differences in food choices, adherence to dietary recommendations and plasma lipid profile in type 2 diabetes—The TOSCA.IT study. Nutr. Metab. Cardiovasc. Dis..

[43] Weickert M.O., Pfeiffer A.F. (2018). Impact of dietary fiber consumption on insulin resistance and the prevention of type 2 diabetes. J. Nutr..

[44] Rienks J., Barbaresko J., Oluwagbemigun K., Schmid M., Nöthlings U. (2018). Polyphenol exposure and risk of type 2 diabetes: Dose-response meta-analyses and systematic review of prospective cohort studies. Am. J. Clin. Nutr..

[45] PREDIMED study investigators (2015). Intake of total polyphenols and some classes of polyphenols is inversely associated with diabetes in elderly people at high cardiovascular disease risk. J. Nutr..

[46] Malik V.S., Li Y., Tobias D.K., Pan A., Hu F.B. (2016). Dietary protein intake and risk of type 2 diabetes in US men and women. Am. J. Epidemiol..

[47] Ye J., Yu Q., Mai W., Liang P., Liu X., Wang Y. (2019). Dietary protein intake and subsequent risk of type 2 diabetes: A dose–response meta-analysis of prospective cohort studies. Acta Diabetol..

[48] Martínez-González M.A., Sanchez-Tainta A., Corella D., Salas-Salvado J., Ros E., Aros F., Gomez-Gracia E., Fiol M., Lamuela-Raventos R.M., Schröder H. (2014). A provegetarian food pattern and reduction in total mortality in the Prevención con Dieta Mediterránea (PREDIMED) study. Am. J. Clin. Nutr..

[49] Mohammadifard N., Sajjadi F., Maghroun M., Alikhasi H., Nilforoushzadeh F., Sarrafzadegan N. (2015). Validation of a simplified food frequency questionnaire for the assessment of dietary habits in Iranian adults: Isfahan Healthy Heart Program, Iran. ARYA Atheroscler..

