# Supplementary material for: Higher Adherence to Plant-Based Diet Lowers Type 2 Diabetes Risk among High and Non-High Cardiovascular Risk Populations: A Cross-Sectional Study in Shanxi, China
Source: Nutrients. 2023 Feb 3;15(3):786. doi: 10.3390/nu15030786 (PMC9920686; doi:10.3390/nu15030786)
Supplement: Supplementary file 1 [file nutrients-15-00786-s001.zip › nutrients-2157517-supplementary.pdf]

Table S1. Classification of food items in the Early Screening and Comprehensive Intervention Program for high-risk Population of Cardiovascular Disease Study

Food groups	Items in the food frequency questionnaire	Frequency	PDI
**Plant foods**			
Vegetables	Fresh vegetables refer to all kinds of fresh or processed vegetables, including all kinds of fresh legumes (e.g. beans, string beans, broad beans, cowpeas, etc.), roots and tubers (e.g. carrots, onions, potatoes, taro, etc.), melons (e.g. winter melon, loofah, pumpkin, cucumber, etc. but excluding watermelon and cantaloupe, etc.), eggplants (e.g. aubergines, peppers, tomatoes, etc.), tender stems, leaf mosses and flowering vegetables (e.g. bok choy, spinach, cabbage, cabbage, celery, bamboo shoots, leeks.)	Almost everyday	5
		≥1 time/week	4
		1-3 time/month	3
		Rarely or never	1
Fruits	A variety of fresh fruits	Almost everyday	5
		≥1 time/week	4
		1-3 time/month	3
		Rarely or never	1
Whole grains	All food crops other than rice and pasta, including millet, maize, sorghum, sweet potatoes etc except potatoes.	Almost everyday	5
		≥1 time/week	4
		1-3 time/month	3
		Rarely or never	1
Legumes	All types of soy-based food products including tofu, dried tofu and beverages including soy milk, etc.	Almost everyday	5
		≥1 time/week	4
		1-3 time/month	3
		Rarely or never	1
Rice	Rice, rice porridge, porridge and rice noodles, etc.	Almost everyday	5
		≥1 time/week	4
		1-3 time/month	3
		Rarely or never	1
Wheat	All kinds of foods made or processed from large wheat flour, such as noodles, steamed buns, steamed buns and flatbread, etc.	Almost everyday	5
		≥1 time/week	4
		1-3 time/month	3
		Rarely or never	1
Salt-preserved vegetable	Refers to vegetable products processed by various methods such as pickling/drying/soaking including various pickles, sauerkraut, kimchi, pickled mustard, preserved vegetables and winter vegetables, etc.	Almost everyday	5
		≥1 time/week	4
		1-3 time/month	3
		Rarely or never	1
**Animal foods**			
Meat	Refers to all kinds of livestock meat and wild animal meat such as pigs, cattle, sheep, rabbits and dogs as well as their corresponding viscera products that are fresh or specially processed, such as pickled/sauced/dried/cured/salted.	Almost everyday	1
		≥1 time/week	3
		1-3 time/month	4
		Rarely or never	5
Poultry	Refers to poultry meat and wild bird meat and their corresponding offal products such as chicken, duck, goose and wild bird meat that	Almost everyday	1
		≥1 time/week	3

	fresh or specially processed poultry meat such as. cured/sauced/sauced/waxy/salted.	1-3 time/month<br>Rarely or never	4<br>5
Egg	Includes all kinds of fresh or specially processed eggs and corresponding egg products, such as duck eggs, goose eggs, quail eggs, preserved eggs, salted eggs, etc.	Almost everyday<br>≥1 time/week<br>1-3 time/month<br>Rarely or never	1<br>3<br>4<br>5
Seafood	All kinds of food aquatic/seafood: all kinds of freshwater and marine fish, shrimps and crabs, as well as all kinds of shellfish and snails, fresh, frozen or specially processed, e.g. cured/sauced.	Almost everyday<br>≥1 time/week<br>1-3 time/month<br>Rarely or never	1<br>3<br>4<br>5
Dairy products	Pure dairy products such as milk, goat's milk, yoghurt, cheese and milk powder.	Almost everyday<br>≥1 time/week<br>1-3 time/month<br>Rarely or never	1<br>3<br>4<br>5

Table S2. Characteristics of vegetables according to quartile of PDI among high cardiovascular risk and non-high cardiovascular risk population

Food type	Group	Frequency	HCRP	non-HCRP
vegetables	Q1	<1-3/month	318(7.6)	1,500(11.7)
		≥1/week	3,855(92.4)	11,292(88.3)
	Q2	<1-3/month	92(2.6)	434(6.3)
		≥ 1/week	3,478(97.4)	6,476(93.7)
	Q3	<1-3/month	69(1.2)	311(3.6)
		≥ 1/week	5,685(98.8)	8,214(96.4)
	Q4	<1-3/month	22(0.6)	79(1.5)
		≥ 1/week	3,736(99.4)	5,133(98.5)

Note: Q, quartile. HCRP, high cardiovascular risk population. Non-HCRP, non-high cardiovascular risk population. The difference among quartiles is tested by chi-square test.

Table S3. Odds ratios (ORs) and 95% confidence intervals (CIs) for type 2 diabetes according to the plant-based diet index after interpolating missing covariates

	T2D (%)	Model 1	Model 2	Model 3
		OR (95% CI)	OR (95% CI)	OR (95% CI)
Total population				
Q1	1516(14.8)	1.00	1.00	1.00
Q2	1439(13.3)	0.89(0.80-0.98) *	0.92(0.83-1.02)	0.92(0.82-1.02)
Q3	1066(11.7)	0.78(0.68-0.88) *	0.82(0.74-0.90) *	0.82(0.74-0.90) *
Q4	628(11.5)	0.76(0.66-0.89) *	0.82(0.73-0.91) *	0.81(0.72-0.91) *
p_{trend}		0.000	0.000	0.000
Non- HCRP				
Q1	1516(14.8)	1.00	1.00	1.00
Q2	1439(13.3)	0.88(0.79-0.98) *	0.93(0.83-1.04)	0.93(0.83-1.04)
Q3	1066(11.7)	0.80(0.69-0.94) *	0.88(0.76-1.01)	0.88(0.76-1.01)
Q4	628(11.5)	0.69(0.60-0.78) *	0.78(0.69-0.88) *	0.78(0.69-0.88) *
p_{trend}		0.000	0.001	0.000
HCRP				
Q1	769(23.3)	1.00	1.00	1.00
Q2	1008(19.8)	0.77(0.68-0.86) *	0.83(0.75-0.93)	0.83(0.74-0.93) *
Q3	1047(17.5)	0.72(0.61-0.86) *	0.82(0.72-0.93) *	0.82(0.72-0.93) *
Q4	702(18)	0.74(0.59-0.92) *	0.81(0.68-0.97) *	0.81(0.68-0.96) *
p_{trend}		0.001	0.004	0.002

Note: HCRP, high cardiovascular risk population. Non-HCRP, non- high cardiovascular risk population. Adjusted for age (year, continuous) and sex , tobacco smoking (never , other), alcohol drinking (≥ 2 days/week , other), physical activity (≥ 1 day/week, other), marital status (married, cohabiting, other), waist circumference (central obesity , normal), geographic region (rural, urban), occupation (farmer, retired, other), education (high school or above, other), household income (< RMB 50,000/≥ RMB 50,000 ）, hypertension (yes, no), hyperlipidemia (yes, no), BMI (<24,24-28, ≥ 28 kg/m^2). * Indicates $p < 0.05$

Table S4. Odds ratios (ORs) and 95% confidence intervals (CIs) for type 2 diabetes according to the plant-based diet index after weighting the data by multinomial propensity score

	Q1	Q2	Q3	Q4
Total population	1.00	0.90(0.83-0.97) *	0.83(0.78-0.90) *	0.82(0.76-0.90) *
Non-HCRP	1.00	0.95(0.87-1.04)	0.92(0.83-1.03)	0.82(0.74-0.92) *
HCRP	1.00	0.83(0.74-0.93) *	0.79(0.71-0.89) *	0.82(0.73-0.92) *

Note: HCRP, high cardiovascular risk population. Non-HCRP, non- high cardiovascular risk population. Adjusted for age (year, continuous) and sex , tobacco smoking (never , other), alcohol drinking (≥ 2 days/week , other), physical activity (≥ 1 day/week, other), marital status (married, cohabiting, other), waist circumference (central obesity, normal), geographic region (rural, urban), occupation (farmer, retired, others), education (high school or above, other), household income (< RMB 50,000, ≥ RMB 50,000), hypertension (yes, no), hyperlipidemia (yes, no), BMI (<24,24-28, ≥ 28 kg/m^2). * Indicates $p < 0.05$.

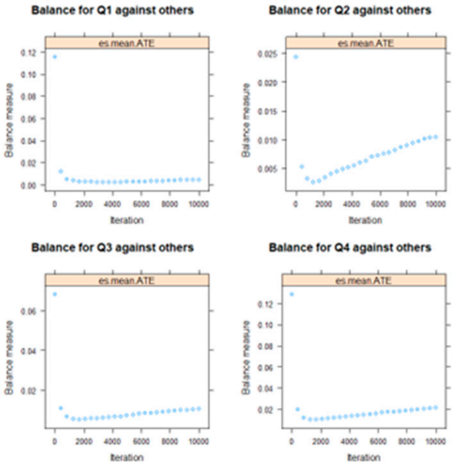

Figure S1. The balance measures of interest at different interactions in the total population

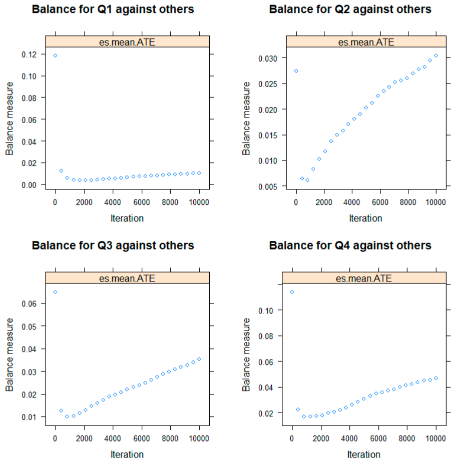

Figure S2. The balance measures of interest at different interactions in the high cardiovascular risk population

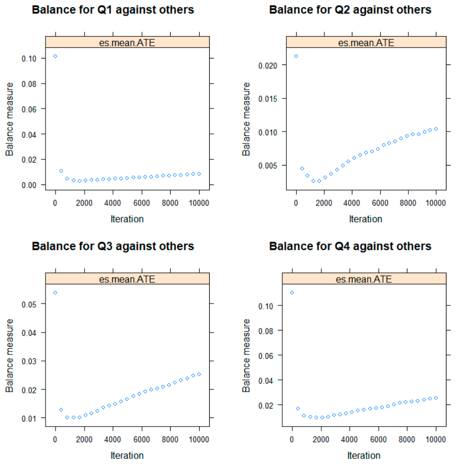

Figure S3. The balance measures of interest at different interactions in the non-high cardiovascular risk population

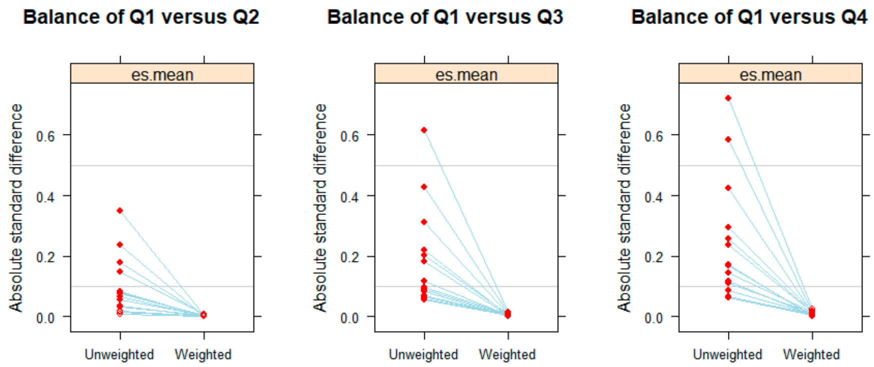

Figure S4. The absolute standardized mean differences (ASMD) of covariates in the total population

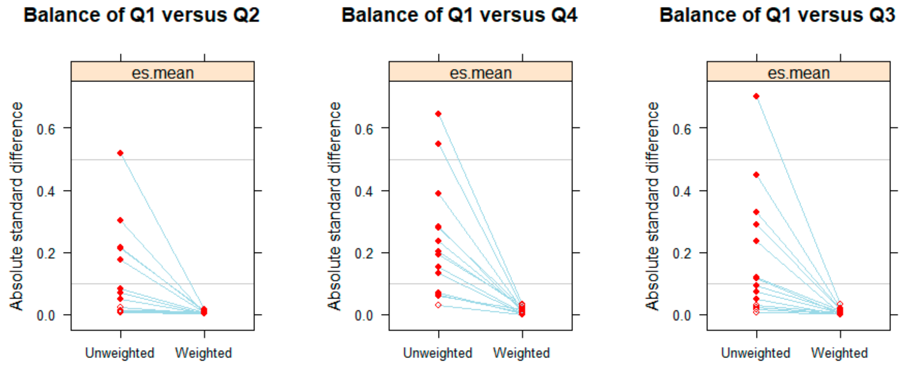

Figure S5. The absolute standardized mean differences (ASMD) of covariates in the high cardiovascular risk population

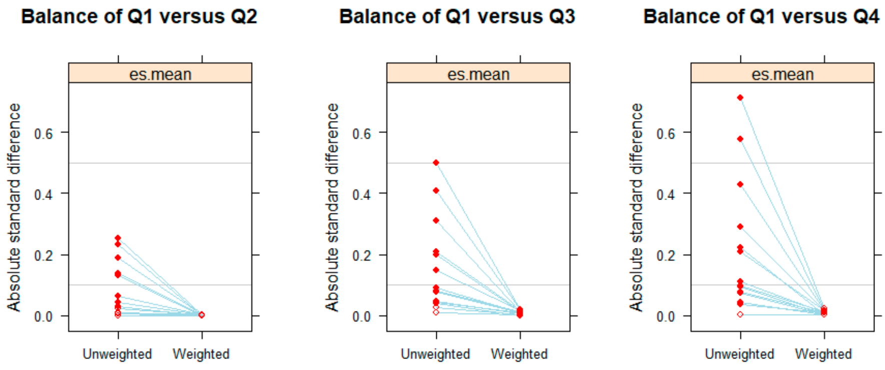

Figure S6. The absolute standardized mean differences (ASMD) of covariates in the non-high cardiovascular risk population
